# A Systematic Review of the Design, Method of Implantation and Early Clinical Outcomes of Transcatheter Tricuspid Prostheses

**DOI:** 10.31083/j.rcm2408231

**Published:** 2023-08-11

**Authors:** Faizus Sazzad, Yinling Zhu, Hwa Liang Leo, Jie Hui Nah, Hui Ying Ang, Chuen Neng Lee, Jimmy Kim Fatt Hon

**Affiliations:** ^1^Department of Surgery, Yong Loo Lin School of Medicine, National University of Singapore, 117597 Singapore, Singapore; ^2^Department of Biomedical Engineering, College of Design and Engineering, National University of Singapore, 117583 Singapore, Singapore; ^3^Department of Cardiac, Thoracic and Vascular Surgery, National University Hospital, 119074 Singapore, Singapore

**Keywords:** tricuspid valve, transcatheter, prosthesis, implantation, systematic review

## Abstract

**Background:**

Intervention for tricuspid regurgitation (TR) tends to happen concurrently with and is addressed during mitral valve surgery. Isolated TR interventions, however, are not unusual and are becoming more common. The purpose of this study was to provide a general overview of the transcatheter tricuspid valve implantation (TTVI) devices, taking into account the several design variations, and to unify the implantation technique, existing clinical results, and potential future directions for TR replacement therapy.

**Methods:**

The major databases, namely Pubmed via Medline, Embase, and Cochrane library, were systematically searched from the date of conception until 10 February 2023, in accordance with the preferred reporting items for systematic reviews and meta-analyses (PRISMA) standards.

**Results:**

Eleven studies were isolated from a total cohort of 5842 publications. All the transcatheter tricuspid prostheses were circular in design yet categorized into annular tricuspid valve implantation (ATVI) and caval valve implantation (CAVI) groups. Bleeding (25.2%), severe access site and vascular issues requiring intervention (5.8%), device migration or embolization (3.6%), and paravalvular leak (38%) are among the early TTVI-related complications that have been observed. The CAVI group experienced 3 of 28 bleeding cases and 2 of 4 device migration cases.

**Conclusions:**

Following the intervention with a transcatheter tricuspid prosthesis, this review discovered an early favorable outcome and a general improvement in heart failure symptoms. However, there was a lot of variation in their design, implantation technique, and early clinical outcomes. Understanding the design variations, difficulty of implantation and learning from this review’s key findings could help with the future development of catheter-based tricuspid valves.

**Systematic Review Registration:**

https://www.crd.york.ac.uk/prospero/display_record.php?ID=CRD42022312142.

## 1. Introduction

The prevalence of tricuspid regurgitation (TR) can vary depending on the population studied and the underlying causes of the disease. TR can affect long-term survival and reduce the quality of life in patients with mitral insufficiency [1, 2, 3, 4]. Surgical intervention of the tricuspid valve, whether repair or replacement, is required to limit the disease progression and right ventricle (RV) dysfunction for the prevention of right heart failure when failed medical therapy can not prevent the TR symptoms [5, 6, 7].

TR intervention is usually concomitant and addressed with mitral valve surgery. However, isolated TR intervention is not uncommon and increasing in numbers [8]. Despite the overall increase in isolated tricuspid intervention rate, the mortality related to the surgery remains high [9]. Transcatheter technology was introduced to overcome the shortcoming in terms of surgical outcomes in heavily comorbid and high-risk surgical candidates, many interventions were carried out for compassionate reasons. Transcatheter tricuspid valve implantation (TTVI), which is a promising option to replace TR, has faced a number of challenges, obstacles, and limitations [10]. A number of TTVI devices in the pipeline, at different stages of their development, show significant differences in terms of design. The variation in the design centers on the method of anchoring the device into the native annulus, which has been popularized as annular tricuspid valve implantation (ATVI), which is an orthotopic method of implantation. The less common technique among the two is caval valve implantation (CAVI) is a heterotrophic method of TTVI into the vena cava [11].

Due to the significant difference that exists in the design, the implantation technique also varies significantly. There is no widespread consensus on the implantation technique. As such, the clinical outcomes from the reported initial clinical trials also differ from one another, keeping a few in common. Therefore, the aim of this review is to provide an overview of the TTVI devices in terms of the variation of their design, harmonize the method of implantation and current clinical outcomes achieved with a glimpse of future perspectives for TR replacement therapies.

## 2. Evolution of Transcatheter Tricuspid Prosthesis

### 2.1 Annular Tricuspid Valve Implantation

After the first TTVI was successfully implanted in an animal in 2005 [12], other devices were being developed and had undergone recent clinical studies. One of the first successful dedicated ATVI in a human native tricuspid valve annulus was reported by Jose L Navia in 2017 [13]. Although the prosthesis was first designed as a surgical implant, later modified as a catheter-based prosthesis, yet using a right mini-thoracotomy approach. One out of two cases of NaviGate valved-stent (NaviGate Cardiac Structures, Inc, Lake Forest, CA, USA) of the first-in-man (FIM) series was a Valve-in-Ring (ViR) procedure [13].

**Fig. 1.**
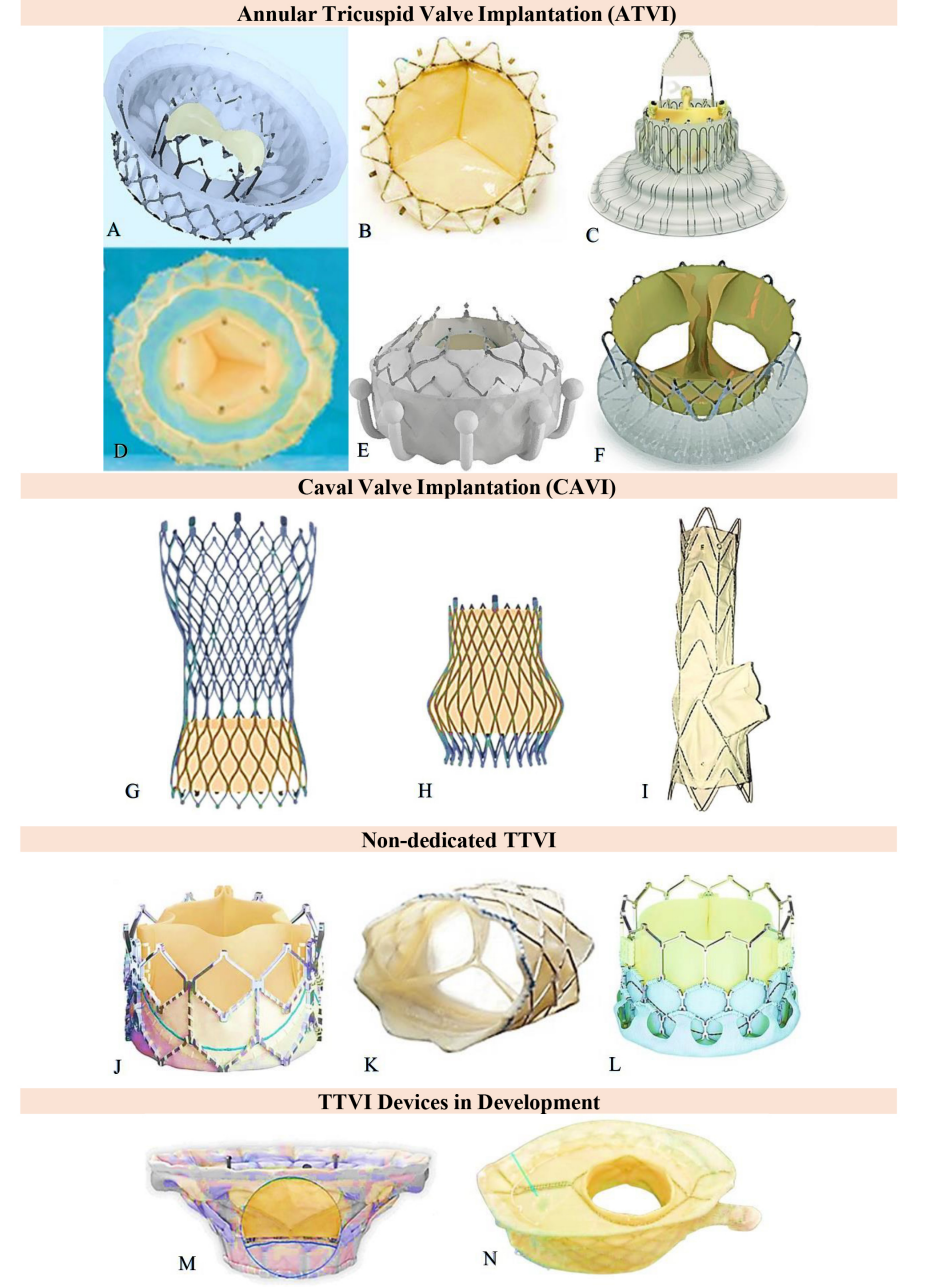
**Transcatheter tricuspid replacement devices**. Annular Tricuspid Valve Implantation (ATVI) devices are (A) Intrepid*, Medtronic Inc. (B) Navigate*, NaviGate Cardiac Systems Ltd. (C) Lux-Valve*, Jenscare Biotech Inc. (D) Topaz#, TriCares Inc. (E) EVOQUE*, Edwards Lifesciences Inc. (F) Trisol*, Trisol Medical; Caval Valve Implantation (CAVI) prosthesis are (G) Tric-SVCǂ, P+F Ltd. (H) Tric-IVCǂ, P+F Ltd. (I) Tricento*, New Valve Tech Ltd.; Non-dedicated devices are (J) Sapien XT*, Edwards Lifesciences Inc. (K) Melody§, MelodyVR, Inc. (L) MyVal€, Meril Life Inc.; Devices in development are (M) Cardiovalve*, Boston Medical Ltd. (N) VDyne¥, VDyne, Inc. *Adopted from Goldberg YH *et al*., (2021) [24]. #Adopted from Straubinger HJ (2021) [20]. ǂAdopted from Sharma NK *et al*., (2021) [25]. §Adopted from Riede FT, & Dähnert I (2012) [22]. €Adopted from Lu F *et al*. (2020) [23]. ¥Adopted from Latib A (2020) [21]. TTVI, transcatheter tricuspid valve implantation.

A few years later, at the Cardiovascular Research Technologies symposium 2020, Vinayak N. Bapat presented the Intrepid valve (Medtronic, Minneapolis, MN, USA) for severe TR FIM case experience [14]. The Intrepid system used was a surrogate of the Intrepid transcatheter mitral valve implantation system [15]. Similarly, EVOQUE (Edwards Lifesciences, Irvine, CA, USA) valve replacement system for TR is identical to their system for transcatheter mitral valve implantation reported its FIM series in 2021 [16]. Meanwhile, the Food and Drug Administration (FDA), United States designated the Cardiovalve system (Cardiovalve Ltd, Or Yehuda, Israel) as a breakthrough device, and it revealed the results of its preclinical testing [17]. The Lux valve (Ningbo Jenscare Biotechnology Co., Ltd, Ningbo, China) also reported its initial clinical success that year [18]. Trisol Valve (Trisol Medical Ltd. Inc. Yokneam, Israel) issued an FIM report later in 2021 to enable high-risk patients to avoid surgery [19]. The first successful use of the Topaz tricuspid heart valve (TRiCares SAS, Paris, France) was also announced in 2021 [20]. Azeem Latib presented the preclinical data for the VDyne Valve (VDYNE, Inc. Maple Grove, MN, USA) at the Transcatheter Cardiovascular Therapeutics Connect (TCTconnect) meeting in 2020 [21], but the VDyne has not yet reported a human use (Fig. 1, Ref. [20, 21, 22, 23, 24, 25]).

### 2.2 Caval Valve Implantation

In a preclinical swine model, Alexander Lauten proposed percutaneous caval transcatheter valve implantation in the superior vena cava and inferior vena cava using a porcine pulmonary valve in 2010 [26]. The FIM application of CAVI was reported by the same group, however, they employed a specially constructed self-expanding valve [27]. It has now been included in the use of Sapien XT/3, an off-label transcatheter aortic valve (Edwards Lifesciences). TricValve (P&F Products Features Vertriebs, Weßling, Germany) was created using Sapien XT/3, and they published their first clinical series in 2018 [28]. A custom-made solution was proposed based on a similar concept, which has reported the successful use of the Tricento (NVT, Hechingen, Germany) valve in humans in the same year [29].

### 2.3 Non-Dedicated Devices

The initial experience with TTVI was restricted to the balloon-expandible Sapien valve Valve-in-Valve (ViV) or ViR procedures (Edwards Lifesciences), done in 2011 and 2014, respectively [30, 31]. The use of a non-dedicated Sapien valve (Edwards Lifesciences) was the first reported ATVI in the native annulus in 2014 [31]. In line with the trend, a pediatric patient had the transcatheter Melody pulmonary valve (MelodyVR, Medtronic, Fridley, MN, USA) implanted as a ViV for TTVI in 2012 [22].

## 3. Materials and Methods

A systematic review was conducted following the Preferred Reporting Items for Systematic Reviews and analyses for systematic review standards [32]. We conducted electronic searches on Medline (via PubMed), Embase, and Cochrane database records from the date of inception to 10 February 2023. On the databases, a repetitive and exhaustive combination of the following ‘Medical Subject Headings’ were used: “Heart Valve Prosthesis Implantation”, “Tricuspid valve insufficiency” and “Heart Valve Prostheses”. By the combination of Medical Subject Headings descriptor relevant keywords, namely, “Transcatheter tricuspid Valve Replacement”, “Transcatheter tricuspid Valve Implantation”, “Transcatheter”, “Tricuspid valve”, “Tricuspid valve surgery”, a complete search statement was generated with additional title/abstract searches in all the databases. An alternative search was carried out on “Clinicaltrial.gov” and “Google Scholar” to verify the authenticity of the extracted information from the primary search. The study protocol was registered with PROSPERO (CRD42022312142) [33].

### 3.1 Study Selection

We have included published articles in English, which mentioned the results of experimental clinical studies in humans reporting first and early clinical trials by using TTVI prosthesis for tricuspid valve disease under appropriate clinical indication. “Transcatheter tricuspid Valve Implantation” was subject to ascertain via “topic”, “title” and “abstract” review during the enrolment process. A combination of the search terms and keywords as per the protocol-defined search strategy was implemented for the appropriate inclusion of a study. All the percutaneous and transcatheter tricuspid valve repair devices were excluded from this study. Hence, this review includes only the transcatheter tricuspid replacement devices. Transcatheter heart valves for other cardiac positions used concomitantly were also beyond the scope of this study as they may produce a confounding effect. Preclinical large animal experiment reports with TTVI and other concomitant cardiac procedures and non-clinical *in vitro* experiments studies were also excluded. Three reviewers screened and assessed the studies independently for inclusion by using the reference software ${\mathrm{EndNote}}^{\text{TM}}$ X9, (Clarivate, Philadelphia, PA, USA). The articles were first screened by their titles and abstracts. The full-text review was performed on articles for studies that have made it through the first stage, cases where a decision cannot be made, or if the reviewers were unable to confirm the relevance of the study for inclusion. A manual search by using the backward snowballing method was also done once further data verification was required. A detailed search strategy has been recorded in **Supplementary Table 1**.

### 3.2 Quality of Evidence 

GRADEpro Guideline Development Tool (McMaster University and Evidence Prime, Ontario, Canada quality of evidence assessment software) was used to evaluate the included studies as illustrated in chapter 11 of the Cochrane handbook of reviews [34]. The quality of the included manuscripts was further assessed for the risk of bias for inclusion by using ReviewManager 5.4 (Cochrane, England software, London, England) [35] in accordance with the guidelines in chapter 8 of the Cochrane handbook of reviews. In our study risk of bias in ramdomized controlled trial was also assessed according to guidelines from the Cochrane handbook. The risk of bias in nonrandomized observational studies was also assessed according to guidelines from the Cochrane handbook, risk of bias was evaluated using the Risk of Bias in Non-randomised Studies of Interventions (ROBINS-I), (Cochrane, London, England) tool [36].

### 3.3 Data Abstraction and Statistical Analyses

The included studies were assessed by two authors independently, and details of the manuscripts were abstracted, including title, authors, year of publication, study type, number of patients, sex, age, TTVI device design description, method of anchoring, route, access of device deployment, periprocedural imaging, and early clinical outcome. The primary outcome measures were the procedural success rate and mortality. The secondary outcome measures were the indication of TTVI, all complications, device failure, all-cause mortality, and specific mortality, which is defined as mortality due to underlying cardiovascular causes. Data synthesis was done utilizing the ReviewManager 5.4 (Cochrane, England software, London, England) [35].

## 4. Results

**Fig. 2.**
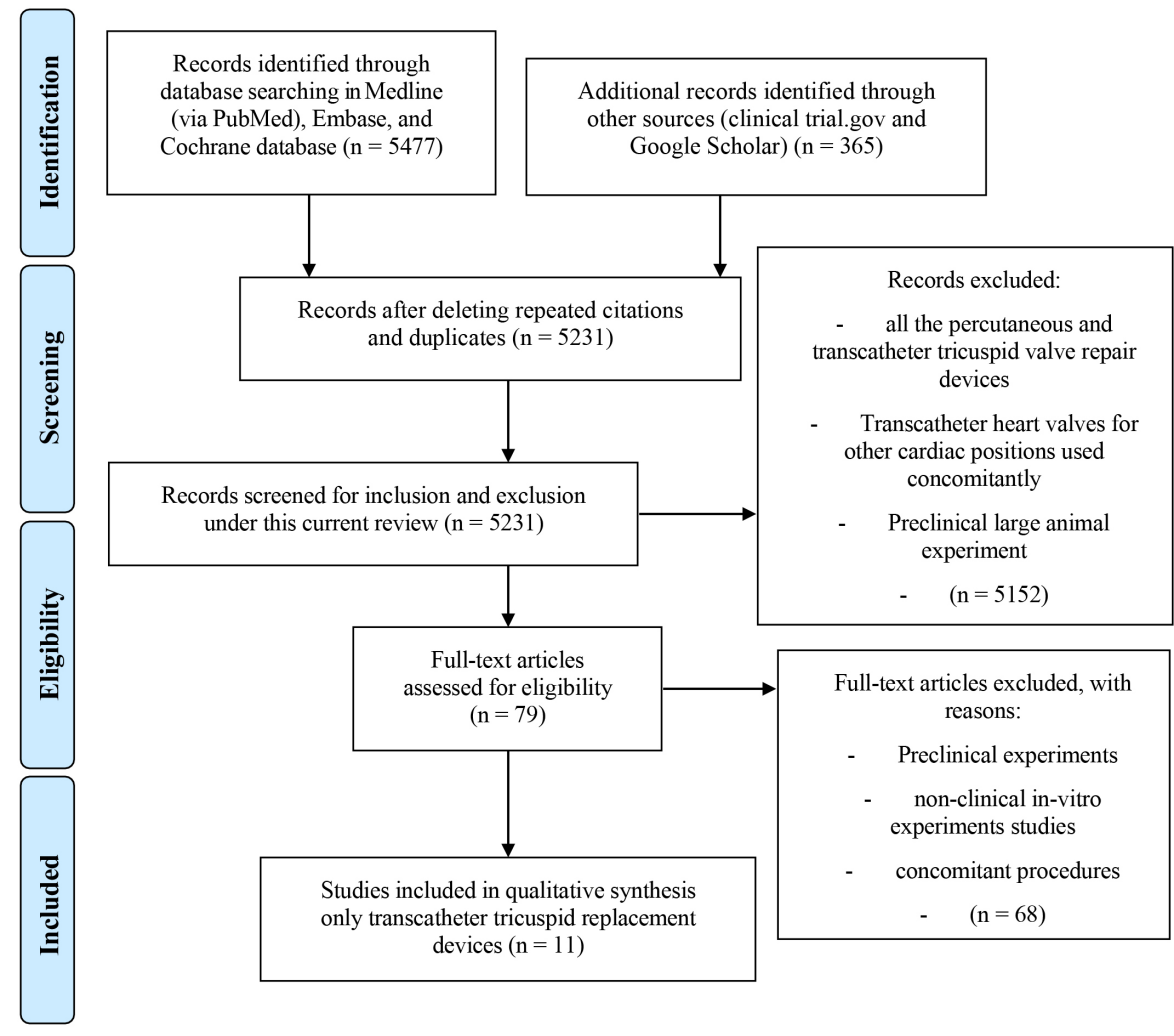
**Preferred reporting items for systematic reviews and meta-analyses (PRISMA)**. The method of stepwise inclusion, assessment, exclusion, and final enrolment of current articles showing, that eleven articles were enrolled in this current review.

Our nonexhaustive systematic search identified a total of 5842 articles which includes 365 publications from alternative sources. After duplicates were removed, 5231 papers remained for review. In the next stage, based on the title and abstract review, irrelevant publications for those that did not satisfy enrolment criteria were excluded, leaving 79 articles for the full-text review. Following the full-text assessment of these articles, 11 articles [14, 16, 19, 20, 22, 28, 29, 37, 38, 39, 40] remained for final review (Fig. 2).

### 4.1 Risk of Bias Assessment

All the included studies were early clinical trials, of which most were the FIM clinical trial. Our risk of bias assessment showed five studies are categorized as high risk of performance bias [14, 19, 20, 22, 40] due to the nature of publication, namely a technical case presentation [14]. Press announcement of the technical success [20], and first case report [20, 22, 40]. All other included studies had an unclear risk of bias as inadequate information was available for blinding and randomization for a conclusion to be made. However, the importance and relevance of these articles were independently assessed by three authors, and the evidence provided by the included studies was found critical/important for inclusion (**Supplementary Table 2**). Characteristics of the included articles have been summarized in Table 1 (Ref. [14, 16, 19, 20, 22, 28, 29, 37, 38, 39, 40]).

**Table 1.** **Characteristics of the included studies**.

Author/Year	Device/Industry	Country	Journal/Source	Patients	Study type	Group	Valve	Follow-up	Clinical trials
Bapat *et al*., 2020 [14]	Intrepid (Medtronic Inc, MN, USA)	USA	CRT presentation	1	FIM	ATVI	Annular	NA	TTVR EFS (NCT04433065)
Kodali *et al*., 2022 [16]	EVOQUE system (Edwards Lifescience, Irvine, CA, USA)	USA	JACC	56	RCT	ATVI	Annular	30 days	TRISCEND II, NCT04482062
Vaturi *et al*., 2021 [19]	Trisol Valve (Trisol Medical Ltd. Inc. Yokneam, Israel)	Israel	JACC	1	FIM	ATVI	Annular	2 weeks	Trisol EFS NCT04905017
Straubinger *et al*., 2021 [20]	Topaz Tricuspid Heart Valve (TriCares SAS, Paris, France)	France	Press release	2	FIM	ATVI	Annular	NA	-
Hahn *et al*., 2020 [37]	NaviGate device (NaviGate Cardiac Structures Inc., Lake Forest, CA, USA)	USA	JACC	30	Case Series	ATVI	Annular	30 days	Transjugular access trial (abandoned)
Sun *et al*., 2021 [38]	LuX-Valve (Jenscare Biotechnology, Ningbo, China)	China	Euro-intervention	6	Case Series	ATVI	Annular	12 months	TRAVEL (NCT04436653)
Lauten *et al*., 2018 [28]	TricValve (P+F Products + Features, Vienna, Austria)	Austria	Circulation Cardiovascular Interventions	25	FIM	CAVI	UniCaval, Bicaval	12 months	TRICUS (NCT03723239) TRICUS Euro (NCT04141137)
Toggweiler *et al*., 2018 [29]	Tricento (New Valve Technology, Hechingen, Germany)	Germany	Euro-intervention	1	FIM	CAVI	Bicaval	3 months	TRICAR (NCT05064514)
Riede *et al*., 2012 [22]	Melody (MelodyVR, Medtronic, Fridley, MN, USA)	USA	Catheterization and Cardiovascular Interventions	1	Case report	Non-dedicated	ViV/ViR	NA	-
Dreger *et al*., 2020 [39]	Edwards Sapien XT/3 (Edwards Lifescience, Irvine, CA, USA)	USA	Euro-intervention	14	RCT	Non-dedicated	Caval	1, 3, 6, 12 months	TRICAVAL, HOVER is ongoing
Karaduman *et al*., 2021 [40]	MyVal THV, (Meril Life Sciences Pvt Ltd, Vapi, Gujarat, India)	India	Annals of Thoracic Surgery	1	Case Report	Non-dedicated	ViV/ViR	1 month	

FIM, first-in-man; CRT, cardiovascular research technologies; ViV, valve-in-valve; ViR, valve-in-ring; JACC, Journal of the American College of Cardiology; RCT, randomized controlled trial; ATVI, annular tricuspid valve implantation; CAVI, caval valve implantation; NA, not applicable; TTVR, transcatheter tricuspid valve replacement; EFS, early feasibility study; MN, Minnesota; CA, California.

### 4.2 TTVI Device Design and Access

All the transcatheter tricuspid prostheses were circular in design yet can be categorized based on the method of implantation (Fig. 1). With the exception of NaviGate (NaviGate Cardiac Structures Inc., Lake Forest, CA, USA), where a transatrial approach was necessary, and a transjugular approach for the same device was abandoned due to procedural complications [37], ATVI is the most prevalent, nearly invariably employing transfemoral access [14, 16, 19, 20, 37, 38]. Although the valve itself is circular, VDyne Valve (VDyne, Inc. Maple Grove, MN, USA) has a varying outer nitinol frame with a “Pop-off” to address afterload mismatch [21]. CAVI devices are also circular, and the caval mounting stent is spanning across the right atrium from superior to inferior vena cava. CAVI devices are classified depending on the location of the valve mounted, but all the available devices are delivered via femoral access. In addition, the Tricento (New Valve Technology, Hechingen, Germany) was a custom-made device [29]. Non-dedicated TTVI devices are made for and in use for other transcatheter therapies (i.e., Melody valve for pulmonary intervention [22]) and also come in a circular shape and are mostly used for ViV or ViR procedures (Table 2, Ref. [14, 16, 19, 20, 22, 28, 29, 37, 38, 39, 40]).

**Table 2.** **Transcatheter tricuspid valve prosthesis design and features**.

Device	Access	Size (mm)	Sheath	Design	Annulus	Mounting	Anchoring	Recapture
A. Annular Tricuspid Valve Implantation (ATVI)								
Intrepid (Medtronic) [14]	Transfemoral	43, 46, 50	35 Fr	Dual-stent system with a self-expanding, trileaflet bovine valve	Circular	Nitinol frame—an outer and inner stent	Radial force and cleats of the outer frame	Yes
EVOQUE (Edwards) [16]	Transfemoral	44, 48	28 Fr	Self-expanding, trileaflet bovine bioprosthetic valve	Circular	Mounted on a nitinol frame	Intra-annular sealing skirt and anchors	-
Trisol Valve (Trisol Medical) [19]	Transjugular	Any annulus size 3	30 Fr	Self-expanding, bileaflet dome-shaped structure	Circular	Conical nitinol stent with a single bovine pericardial dome	Anchored by the axial force applied	Yes and retrievable
Topaz (TriCares) [20]	Transfemoral	-	-	Bovine pericardial self-expanding valve	Circular	Nitinol frame	-	-
NaviGate (NaviGate Cardiac) [37]	Transatrial	36–52 (4 sizes)	42 Fr hydro	Self-expanding trileaflet equine pericardial valve	Circular	Tapered nitinol stent with polyester microfiber on atrial winglets	Anchored with 12 tynes on the ventricular side and 12 atrial winglets	-
LuX-Valve (Jenscare Biotech) [38]	Transatrial	Outer-50, 60, 70 Inner-26, 28	32 Fr	Self-expanding bovine pericardial tissue valve and does not rely on radial forces	Circular	Mounted on a nitinol stent annulus covered by polyethylene terephthalate	Two anterior leaflet clampers and an anchor attach to the septum	-
B. Caval valve implantation (CAVI)								
TricValve (P + F) [28]	Transfemoral	S-25, 29 I-31, 35	24 Fr	Two self-expanding bioprostheses & bovine pericardium leaflets	Both SVC and IVC	On a nitinol stent	Radial force, SVC (long skirt), IVC (short skirt)	-
Tricento (New Valve Tech) [29]	Transfemoral	Up to 48	24 Fr	Custom-made self-expanding bicuspid valved stent (porcine pericardium)	From IVC to SVC	13.5 cm covered stent, with non-covered segment for hepatic vein	Radial force-landing zones in SVC and IVC	-
C. Non-dedicated devices								
Melody (MelodyVR) [22]	Transfemoral	18, 20, 22	22 Fr	Trileaflet, tunnel shaped, made of a bovine jugular vein valve	ATVI-ViV/ViR	Platinum-iridium frame	By deploying a stent in a pre-existing valve/ring	None
Edwards Sapien XT/3 (Edwards) [39]	Transfemoral	20, 23, 26, 29	14 Fr or 16 Fr	Trileaflet bovine pericardial valve is attached to a balloon expandable. Sapien in stent	Only IVC CAVI	Cobalt-chromium frame with a polyethylene terephthalate skirt	Anchoring is only obtained by deploying a stent in the IVC	-
MyVal (Meril Life) [40]	Transfemoral	20–32 (9 sizes)	14 Fr Python	Circular trileaflet bovine pericardium	ATVI- ViV/ViR	Cobalt alloy frame	Anchoring is achieved by radial force	-
D. Devices in development								
CardioValve (Boston Medical)	Transfemoral	M/45, L/50, XL/55	28 Fr	Self-expanding, tri-leaflet bovine bioprosthetic valve	Circular	Mounted on a nitinol frame	Anchoring is achieved via leaflet grasping and an atrial flange	-
VDyne Valve (VDyne, Inc.)	Transfemoral	Outer 140–180 (5 sizes)	28 Fr Side delivery	Porcine pericardium, trileaflet, self-expandable valve	Matched shape	Varying outer nitinol frame with a “Pop-off” for afterload mismatch	RVOT Tab, proximal loop and by oversizing	Yes and retrievable

SVC, superiror vena cava; IVC, inferiror vena cava; ViV, valve-in-valve; ATVI, annular tricuspid valve implantation; ViR, valve-in-ring; RVOT, right ventricle outflow tract.

### 4.3 Method of Implantation 

The anatomical landmarks of the tricuspid valve and right heart are commonly assessed in the periprocedural period. TTVI devices are implanted to treat TR, needing the designs to accommodate a non-calcified construct that is both dynamic and D-shaped in one plane and saddle-shaped overall. On top of proper anchoring, TTVI devices need to conform to the native tricuspid annulus to apply the proper sealing required to prevent leakage through the interface of the valve stent and the native annulus, also as known as paravalvular leakage. In our study, we found a variety of different anchoring techniques have been proposed: using tethers to achieve counteracting axial forces (i.e., EVOQUE system (Edwards Lifesciences, Irvine, CA, USA) [16]); native leaflet grasping to fixate the prosthesis in place (i.e., CardioValve (Boston Medical, Shrewsbury, MA, USA) [17]); docking systems to allow radial forces sufficient enough for fixation (i.e., Trisol Valve (Trisol Medical Ltd. Inc. Yokneam, Israel) [19]). However, most CAVI devices are kept in situ by radial force to fix them into the mounting stent [28, 29] (Table 2).

### 4.4 Early Clinical Outcome

Only 2 of the 11 TTVI systems analyzed in this review, the Edwards Sapien XT/3 (Edwards Lifesciences, Irvine, CA, USA) [39] and the Edwards EVOQUE system [16], were recommended for use in clinical trials. One valve system, Melody (MelodyVR, Medtronic, Fridley, MN, USA) [22], was used off-label, while the other nine were indicated for compassionate use in FIM case reports and series. All patients were high-risk cases with moderate to severe TR that was deemed to be of high surgical risk. The baseline characteristics of TTVI recipients are summarized in **Supplementary Table 3**. The mean age of patients was 76.1 years old, with one case study included a 12-year-old pediatric patient. 68.8% of the recipients were women, and the mean weight was 74.4 kg. Most patients (88.1%) were New York Heart Association (NYHA) class III or IV. The mean calculated European System for Cardiac Operative Risk Evaluation (EuroSCORE) II was 9.86%, ranging from 5.6% to 18.2%. A history of cardiac pathology, including atrial fibrillation (89.1%), and past valvular interventions (38.1%), was not uncommon. Other comorbidities such as chronic kidney disease (65.5%), diabetes mellitus (30.6%), and prior cerebrovascular events (17.1%) were also present in the studied populations. The average pulmonary artery systolic pressure was 39.2 mmHg (24.5–74 mmHg), and the mean left ventricular ejection fraction was 54.2% (51–65%). 9.4% of the patients had a primary TR diagnosis, 11.8% had a mixed pathology, and 76.4% of the patients had secondary TR. Transfemoral access for TTVI was used in 72.2% of patients, and the typical implantation time was 85.8 minutes (9.1–210 minutes). The majority of patients had their valves installed successfully, with a reported procedural success rate of 93.2%. Patients spent an average of 6.6 days in the hospital, which was the median length of stay (1–13.5 days). The procedural outcome has been summarized in Table 3 (Ref. [14, 16, 19, 20, 22, 28, 29, 37, 38, 39, 40]).

**Table 3.** **Early procedural clinical outcome of the transcatheter tricuspid implants**.

/	NYHA class n (%)	EuroSCORE II (%)	Mean implantation time, min	Procedural success, n (%)	Median hospital length of stay, days	All cause mortality, n (%)	Cardiovascular mortality, n (%)	Conversion to surgery, n (%)
Intrepid (Medtronic) [14]	II/III 1 (100)	15.54	–	–	6	–	–	–
EVOQUE (Edwards) [16]	III/IV 49 (87.5)	5.6 ± 4.9	70.1 ± 31.5	54 (96.4)	3 (1.0–25.0)	2 (3.6)	Device migration and resultant RV failure 1 (1.8)	–
Trisol (Trisol Medical) [19]	–	–	210	–	6	–	–	–
Topaz (TriCares) [20]	–	–	14 ± 2	–	4 (4–4)	–	–	–
NaviGate (NaviGate Cardiac) [37]	I 0 (0)	11.1 (7.16–14.11)	0	26 (87)	13.5 (7–22)	3 (10)	1	2/30 (7)
	II 4 (14)							
	III 16 (57)							
	IV 8 (29)							
LuX-Valve (Jenscare Biotech) [38]	III 3	7.9 (6.4–9.2)	9.7 (6.2–13.6)	6 (100)	8 (6–11)	–	–	–
	IV 3							
TricValve (P + F) [28]	III 7 (28)	18.2 ± 12.9	–	23 (92)	–	6 (24)	–	Migration of SVC prosthesis 1, Migration of IVC prosthesis into RA 1
	IV 18 (72)							
Tricento (New Valve Tech) [29]	IV 1 (100)	–	45	–	–	–	–	–
Melody (MelodyVR) [22]	–	–	9.1	–	4	–	–	–
Sapien XT/3 (Edwards) [39]	I 0 (0)	–	–	–	–	3	–	Cardiac tamponade due to stent migrate 2 (14.3), Valve dislocations 2 (14.3)
	II 2 (14)							
	III 12 (86)							
	IV 0 (0)							
MyVal (Meril Life) [40]	III 1 (100)	–	–	–	1	–	–	–

NYHA, New York Heart Association; RV, right ventricle; SVC, superior vena cava; IVC, inferior vena cava; RA, right atrium.

### 4.5 Post-Procedural Complications

Results from the procedure and the post-procedure have been compiled in **Supplementary Table 4**. Early TTVI-related complications reported include bleeding (25.2%), major access site and vascular complications requiring intervention (5.8%), device migration or embolization (3.6%), and paravalvular leak (38%). 3 of 28 bleeding cases and 2 of 4 device migration cases occurred in the CAVI group. 8 (11.6%) patients required conversion to open surgery, of which six were from the CAVI group. Of these 6 cases, reported indications included valve migration (n = 2), valve-dislocations (n = 2), and cardiac tamponade from stent migration (n = 2). Cardiovascular mortality and all-cause mortality post-procedure was 2.3% and 11.2%, respectively. Notably, out of the 14 deaths post-procedure, nine occurred in the CAVI group.

### 4.6 Follow-Up Results

Follow-up results are available for 7 out of 11 of the TTVI systems. Follow-up duration varied greatly among the studies, with a median of 2 months and ranging from 2 weeks to 12 months. 28 out of 62 (45.2%) all-cause mortality were reported from post-procedure till to follow-up. Parameters like mean left ventricular ejection fraction improved to 57.2% (55–70%), and mean pulmonary artery systolic pressure reduced to 33.9 mmHg (32.2–37.0 mmHg). There was an overall improvement in the NYHA class of patients. Of the four studies that reported NYHA class at follow-up, EVOQUE (78.8%) (Edwards Lifesciences, Irvine, CA, USA) [16], GATE (72.0%) (NaviGate Cardiac Structures Inc., Lake Forest, CA, USA) [37], and TricValve (52.7%) (P+F Products + Features, Vienna, Austria) [28] showed that a majority of their patients were NYHA class I or II at follow-up, as compared to class III/IV preoperatively. The remaining study, Edwards Sapien XT (Edwards Lifesciences, Irvine, CA, USA) [39], reported that 63% of their patients improved by 1 NYHA class.

## 5. Discussion

The TTVI prostheses design, their method of implantation, and a compiled summary of the early clinical outcomes have all been discussed in this systematic review. TTVI carries sizable surgical risk for the vast majority of patients, however, the results show that TTVI has potential for growth [8, 9]. Although all transcatheter tricuspid prostheses were round in shape, they were divided into several groups according to how they were implanted. ATVI was the most prevalent method and was nearly always accessed via transfemoral routes [14, 15, 16].

Due to the complicated structure of the tricuspid valve, the current focus of ATVI was the anchoring mechanism. The predominant anchor force was still the annular force resulting from the oversized stent diameter relative to the annular size. The VDyne Valve includes a varying outer nitinol frame with an extra grasping even though the valve body is round [21]. The Lux-Valve had a “bird tongue-shaped” anchoring needle with tiny splinters that would pierce the inner layer of the ventricular heart muscle to prevent migration and relieve strain on the annular ring [23, 41]. NaviGate possesses artery winglets that address the annular ring and right ventricle tines that catch the chordae tendineae gap [13]. Topaz used a “stent within a stent” arrangement. The external stent had a low stiffness and high flexibility to adapt and align with the annular ring, whereas the internal stent had a strong stiffness to ensure the bioprosthesis sutured to it continued to operate [20]. Notable was the fact that Trisol had revolutionized the leaflet. It was a single leaflet attached partially to the stent orifice. When the valve was opened, the leaflet’s free edge collapsed toward the center. It allows for partial reflux, and the leaflet’s dome-shaped design collects a certain volume of blood during systole before returning some or all of it during diastole.

In addition to being circular, CAVI devices also have a caval mounting stent that crosses the right atrium from superior to inferior vena cava. All of the available CAVI devices are implanted via femoral access and are categorized based on where the valve is positioned. ATVI appears to offer a more favorable prognosis than CAVI. CAVI does, however provide an intriguing option for patients with pre-existing pacemakers. In the case of a dual chamber pacemaker, the tricuspid valve must be traversed by the right ventricle lead. This makes anchoring more difficult if annular tricuspid valve implantation is required. CAVI thus refers to the heterotopic placement of a valve in the inferior cava alone or in conjunction with a second valve in the superior cava in order to contain the regurgitant jet from a failing tricuspid valve within the right atrium [39]. Therefore, it is fair to assume that this arrangement will cause an increase in pressure within the right atrium, thereby limiting regurgitation through the tricuspid valve. By lowering chronic volume overload, this strategy protects the hepatic and renal veins, hence likely to relieve right heart congestion. That’s been reflected in the results of the TricValve system in patients with severe TR after six months showed that 97% of cases had technical success and that there had been considerable improvements in functional status and quality of life measures [42].

The Melody valve for pulmonary intervention [22] is an example of a non-dedicated TTVI device that is made for and utilized for different transcatheter therapies. These devices are also circular in shape and are typically used for ViV or ViR procedures. NaviGate, a transjugular route for the device implantation, was abandoned due to procedural difficulties, necessitating a transatrial (right thoracotomy) approach instead.

It is important to note that, despite being within normal limits in a routine peri-procedural transesophageal echocardiography (TEE), abnormally increased residual transvalvular gradients are measured in transthoracic echocardiography (TTE) before discharge or at 30-days in 60–80% of patients treated successfully with ViV for a failed bioprosthetic surgical heart valve in a mitral or tricuspid position [43]. A few *in vitro* investigations found that the changed trans-valvular flow characteristics were significantly impacted by the actual transcatheter heart valve frame geometry, i.e., eccentricity/non-round shape and under-expansion [44, 45]. For both de novo and ViV transcatheter replacements, the pattern of the restored blood flow and the long-term results of valve deployment are determined by the actual 3-dimensional expansion of the transcatheter heart valve (THV) stent frame [45]. There is no reliable peri-procedural indicator for the actual THV expansion because it deviates significantly from the nominal value. For direct peri-procedural measurement of THV stent frame and leaflet geometry, large field-of-view intravascular ultrasound (IVUS) gives a distinctive tomographic perspective [46]. A large field-of-view IVUS might take the place of TEE and intra-cardiac echocardiography for the most precise procedural guidance of any THV replacement, IVUS.

According to the clinical findings and follow-up statistics, the median duration of stay for patients was 6.6 days, and an estimated 93.2% of patients had their valves implanted successfully. The results are positive and closely resemble the trailblazing results of transcatheter aortic valve implantation [42]. There was an overall improvement reported in this review, at least by 1 NYHA class in 7–11 months post-procedure follow-up. However, several other transcatheter tricuspid repair devices are currently available, and some are in clinical use [47]. It’s crucial to keep in mind that these technologies are still being tested and improved, and clinical results may differ based on patient selection, operator skill, and unique patient features. The decision to employ one of these devices over another is often based on the individual circumstances of each patient, taking into account things like the degree of tricuspid regurgitation, the patient’s symptoms, and anatomical compatibility. A true comparison between repair and replacement devices could not be made because repair devices were not included in this evaluation.

### Limitations

Despite the advantages of a systematic investigation, our current review has a number of drawbacks. First off, due to their observational character, the FIM studies we included in our systematic review have built-in biases like selection bias. Additionally, some centers might have had financial constraints or concerns that influenced their choice of intervention. The lack of randomized controlled trials comparing ATVI and CAVI procedures in the literature was another obstacle. In order to provide more robust evidence for the best treatment plan for TTVI, further research, and analysis must be done with a larger patient cohort.

## 6. Conclusions

The majority of the devices, according to the current review, are circular and are inserted and secured utilizing radial forces. Early clinical data showed effectively implanted valves. The outcomes are encouraging and strikingly reflect the ground-breaking outcomes of transcatheter aortic valve implantation. This review found an overall improvement in heart failure symptoms on follow-up. Understanding the history of the tricuspid bioprosthesis first-in-man clinical trial, the design and development of a transcatheter tricuspid valve, the performance and early results of the valve, and the significance of the clinical data necessary to start a “de Novo” transcatheter implant.

## Data Availability

All data generated or analyzed during this study are included in this published article.

## Abbreviations

ATVI, annular tricuspid valve implantation; CAVI, caval valve implantation; FIM, first-in-man; IVUS, intravascular ultrasound; NYHA, New York Heart Association; TR, tricuspid regurgitation; TEE, transesophageal echocardiography; THV, transcatheter heart valve; TTVI, transcatheter tricuspid valve implantation; ViR, valve-in-ring; ViV, valve-in-valve.

## Supplementary Material

Supplementary material associated with this article can be found, in the online version, at https://doi.org/10.31083/j.rcm2408231.
