# Supplementary material for: A Systematic Review of the Design, Method of Implantation and Early Clinical Outcomes of Transcatheter Tricuspid Prostheses
Source: Rev Cardiovasc Med. 2023 Aug 11;24(8):231. doi: 10.31083/j.rcm2408231 (PMC11262438; doi:10.31083/j.rcm2408231)
Supplement: Supplementary file 1 [file 2153-8174-24-8-231-s1.zip › Supplementary Material.docx]

**Supplementary Table 1: Search strategy**

| **1. PubMed 1946 to date – new interface,** see <https://pubmed.ncbi.nlm.nih.gov/advanced/>  Search completed on 22/2/2023 | | | |
| --- | --- | --- | --- |
| Statement Number | Search Statement | | Results  22/2/23 |
| 1 | ("Heart Valve Prosthesis Implantation"[Mesh]) OR ((Transcatheter tricuspid Valve Implantation[Title/Abstract]) OR (Transcatheter tricuspid Valve Replacement[Title/Abstract]))) | | 32,725 |
| 2 | ("Tricuspid valve insufficiency"[Mesh]) OR ((((((Tricuspid Valve Regurgitation[Title/Abstract]) OR (Insufficiency, Tricuspid Valve [Title/Abstract])) OR (Valve Insufficiency, Tricuspid[Title/Abstract])) OR (Regurgitation, Tricuspid[Title/Abstract])) | | 7,118 |
| 3 | ("Heart Valve Prostheses"[Mesh]) OR ((((Valve Prosthesis, Heart) OR (Prosthesis, Heart Valve)) OR (Prosthesis, Cardiac Valve)) OR (Cardiac Valve Prosthesis)) | | 57,890 |
| 4 | 1 and 2 and 3 | | 1207 |
| **2. Embase 1974 to 2023, February 02**  Search completed on 22/02/2023 | | | |
| Statement Number | Search Statement | | Results  22/2/23 |
| #1 | 'randomized controlled trial'/exp OR 'randomized controlled trial' OR 'rct' | | 953,643 |
| #2 | 'cohort study' OR 'cohort' | | 1,315,815 |
| #3 | #1 OR #2 | | 2,218,752 |
| #4 | 'heart surgery' OR 'cardiac surgery' OR 'heart surg*' OR 'cardiac surg*' | | 206,481 |
| #5 | 'cardiovascular surgical procedure' OR 'heart valve prosthesis implantation' OR 'valvular surgery' | | 2,155 |
| #6 | 'valve surg*' OR 'tricuspid valve surg*' 'valvular surg*' OR 'cardiovascular surg*' | | 161,356 |
| #7 | #4 OR #5 OR #6 | | 162,567 |
| #8 | 'tricuspid insufficiency' OR 'tricuspid regurgitation'/exp OR 'tr' | | 200,022 |
| #9 | 'transcatheter' | | 66,070 |
| #10 | #8 OR # 9 | | 356,433 |
| #11 | #3 AND #7 AND #10 | | 4,224 |
| **3. Cochrane Library**  Search completed on 24/02/2023 | | | |
| Statement Number | Search statement | | Results found 24/02/2023 |
| #1 | MeSH descriptor: [Heart Valve Prosthesis] explode all trees | | 1442 |
| #2 | (Transcatheter Tricuspid Valve Replacement):ti,ab,kw OR (Transcatheter Tricuspid Valve Implantation):ti,ab,kw | | 36 |
| #3 | #1 OR #2 | | 1463 |
| #4 | MeSH descriptor: [Tricuspid valve insufficiency] explode all trees | | 126 |
| #5 | (Transcatheter tricuspid Valve Implantation):ti,ab,kw OR (Transcatheter tricuspid Valve Replacement):ti,ab,kw | | 36 |
| #6 | #4 OR #5 | | 156 |
| #7 | #3 AND #6 | | 46 |
| **4.** **ClinicalTrials.gov**  Search completed on 24/02/2023 | | | |
| Condition or disease | AND | AND | Studies found 24/02/2023 |
| Cardiovascular Surgical Procedures | Tricuspid valve | Transcatheter | 39 |
| **5. Google Scholar**  Search completed on 24/02/2023 | | | |
| Condition or disease | AND Other terms | AND Intervention/treatment | Studies found 24/02/2023 |
| Transcatheter tricuspid Valve Implantation | Transcatheter tricuspid Valve Replacement | TTVI | 326 |

**Supplementary Table 2: Risk of bias assessment in GradePro**

| **Certainty assessment** | | | | | | | **№ of patients** | | **Effect** | | **Certainty** | **Importance** |
| --- | --- | --- | --- | --- | --- | --- | --- | --- | --- | --- | --- | --- |
| **№ of studies** | **Study design** | **Risk of bias** | **Inconsistency** | **Indirectness** | **Imprecision** | **Other considerations** | **ATVI** | **CAVI** | **Relative (95% CI)** | **Absolute (95% CI)** |  |  |
| **Procedural Success** | | | | | | | | | | | | |
| 4 | observational studies | not serious | not serious | not serious | serious^a^ | none | 86/138 (62.3%) | - | - | - | ⨁⨁⨁◯ Moderate | CRITICAL |
| **Device Design** | | | | | | | | | | | | |
| 8 | observational studies | not serious^a^ | not serious | not serious | serious^a^ | none | 98/138 (71.0%) | not pooled | not pooled | see comment | ⨁⨁⨁◯ Moderate | CRITICAL |
| **All Cause Mortality** | | | | | | | | | | | | |
| 5 | observational studies | not serious | not serious | not serious | serious^a^ | none | 4/138 (2.9%) | - | - | - | ⨁⨁⨁◯ Moderate | CRITICAL |
| **Method of implantation** | | | | | | | | | | | | |
| 11 | observational studies | serious^a^ | not serious | not serious | serious^a^ | none | 98/138 (71.0%) | - | - | - | ⨁⨁◯◯ Low | IMPORTANT |

**CI:** confidence interval

#### Explanations

a. Observational Study

**Supplementary Table 3. Baseline patients characteristics**

| **Device** | **Age (Years)** | **Female**  **n(%)** | **Weight (Kg)** | **Pathology, n(%)** | **LVEF (%)** | **PASP, mmHg** | **CKD, n(%)** | **DM, n(%)** | **CVD (TIA/ Stroke) n(%)** | **Prior valve surgery n(%)** | **Structural anomaly n(%)** | **AF,**  **n(%)** |
| --- | --- | --- | --- | --- | --- | --- | --- | --- | --- | --- | --- | --- |
| Intrepid [14] | 86 | 0 (0) | 68 | - | - | - | - | - | - | - | - | - |
| Evoque [16] | 79.3 ± 7.7 | 43 (76.8) | 73.7 ±  18.6 | Primary 6 (10.7), Secondary 38 (67.9), Mixed 10 (17.9),  Pacer related 1 (1.8), Indeterminate 1 (1.8) | 53.4 ± 10.2 | 40.1 ± 10.5 | 37 (66.1) | 12 (21.4) | 15 (26.8) | 22 (39.3) | _- | 51 (91.1) |
| TriSol [19] | 71 | 1 | - | Primary 1 (100) | 65 | 74 | 0 | - | - | Aortic prosthesis 1 (100) | Mitral valve stenosis 1 (100) | - |
| Topaz [20] | 78 ± 8 | 2 | - | - | - | - | - | - | - | - | - | - |
| NaviGate [34] | 78 (70-80) | 17 (56) | - | - | 55 (46-60) | 38 (30-55) | 19 (63) | 11 (37) | 2 (7) | 12 (40) | Mitral valve stenosis 2(7) Pulmonary regurgitation 1 (3) | 27 (90) |
| LuX-Valve [35] | 56 (53-60) | 6 (100) | _ | _ | 62 (55-70) | 24.5 (19-32) | _ | _ | _ | Mitral 4 Mitral and Aortic 2 | _ | 4 (66.6) |
| Tric [24] | 73.9 ± 0.7 | 13 (52) | 78.1 ± 11.6 | Primary 1 (4) Secondary 24 (96) | 51 ± 15 (15-74) | 41.0 ± 13.9 | _ | 11 (44) | 2 (8) | TAVR 1,  TPVR 2 MitraClip 2 | _ | _ |
| Tricento [25] | 74 | 1 (100) | _ | Secondary 1 (100) | _ | _ | 1 (100) | _ | _ | _ | Calcified MV | _ |
| Melody [28] | 12 | _ | 30 | Secondary 1 (100) | _ | _ | _ | _ | _ | ASD repair, Tricuspid 1(100) | _ | _ |
| Sapien XT [36] | 77 (68.2-82.0) | 12 (86) | _ | _ | 56.4 ± 6.4 | 39.0 (33.5-55.5) | _ | _ | _ | 3 (21) | _ | _ |
| MyVal [37] | 59 | 0 (0) | _ | Secondary 1 (100) | 60 | _ | _ | _ | _ | Tricuspid 1(100) | Ebstein anomaly 1 (100) | _ |

**Supplementary Table 4. Post procedure outcomes and follow up**

| **Device** | **Bleeding, n (%)** | **Major access site and vascular complications requiring intervention, n (%)** | **Device mirgration/ embolisation, n (%)** | **Paravalvular leak, n (%)** | **Conversion to surgery, n (%)** |
| --- | --- | --- | --- | --- | --- |
| Intrepid [14] | _ | _ | _ | _ | _ |
| Evoque [16] | Total 15 (26.8) Fatal 0 Life threatening 0 Extensive 7 (12.5) Major 8 (14.3) | 1 (1.8) | 1 | _ | _ |
| TriSol [19] | _ | _ | _ | _ | _ |
| Topaz [20] | _ | _ | _ | _ | _ |
| NaviGate [34] | 10 (33) | 4 (13) | 1 | 13/24 (54) | 2/30 (7) |
| LuX-Valve [35] | _ | _ | _ | Mild 3 Moderate 1 | _ |
| Tric [24] | 3 (12) | _ | 2 (8) | _ | Migration of SVC prosthesis 1,  Migration of IVC prosthesis into RA 1 |
| Tricento [25] | _ | _ | _ | _ | _ |
| Melody [28] | _ | _ | _ | _ | _ |
| Sapien [36] | _ | _ | _ | 2 (14.3) | Cardiac tamponade due to stent migrate 2 (14.3), Valve dislocations 2 (14.3) |
| MyVal [37] | _ | _ | _ | _ | _ |
